# Effects of high-intensity interval training and moderate-intensity continuous training on mitochondrial dynamics in human skeletal muscle

**DOI:** 10.3389/fphys.2025.1554222

**Published:** 2025-04-17

**Authors:** Yuqing Li, Wanjun Zhao, Qi Yang

**Affiliations:** ^1^ Orthopedic Department, Hunan Children’s Hospital (The Affiliated Children’s Hospital of Xiangya School of Medicine, Central South University), Changsha, Hunan, China; ^2^ Hunan Provincial Key Laboratory of Pediatric Orthopedics, Changsha, Hunan, China; ^3^ The School of Pediatrics, University of South China, Changsha, Hunan, China; ^4^ Otolaryngology-Head and Neck Surgery Department, Shandong Provincial ENT Hospital, Shandong University, Jinan, Shandong, China; ^5^ Physical Education and Sports Training School, Hunan Provincial Sports Vocational College, Changsha, Hunan, China

**Keywords:** high-intensity interval training, moderate-intensity interval training, mitochondrial dynamics, mitochondrial network remodeling, skeletal muscle

## Abstract

Exercise and physical activity confer health advantages, in part, by enhancing skeletal muscle mitochondrial respiratory function. The objective of this study is to analyze the impacts of high-intensity interval training (HIIT) and moderate-intensity continuous training (MICT) on the dynamics and functionality of the mitochondrial network within skeletal muscle. 20 young male participants were assigned to either HIIT or MICT group. Initial assessments of exercise-related indicators were conducted, followed by skeletal muscle biopsies from the vastus lateralis before, 1 day after, and 6 weeks post-experiment. We utilized multi-dimensional myofiber imaging to analyze mitochondrial morphology and arrangement, and assessed citrate synthase activity, complex I activity, and dynamics-related mRNA. Both training modalities increased VO_2max_, W_max_, citrate synthase and complex I activities, mitochondrial content, and volume density, though the changes differed between the two groups. 6 weeks training induced remodeling of the mitochondrial network within skeletal muscle. Before training, the network appeared sparse and punctate. After MICT, it adopted a grid-like structure with partially robust longitudinal connections. In contrast, HIIT resulted in a less obvious grid structure but showed a stronger longitudinally oriented network. Training also increased mRNA expression of mitochondrial fusion proteins and decreased fission protein expression, with these effects being more pronounced in HIIT. Similarly, peroxisome proliferator-activated receptor γ coactivator 1-alpha mRNA expression showed a comparable trend, though the changes differed between 1 day and 6 weeks of training. In conclusion, HIIT and MICT induce distinct mitochondrial adaptation in skeletal muscle, reflected in different network remodeling and molecular pathways. These findings may be due to HIIT’s more pronounced effect on mitochondrial dynamics or respiratory function, but the study has only conducted preliminary observational experiments and further evidence is required for confirmation.

## 1 Introduction

Mitochondria in skeletal muscle form a dynamic, interconnected network that constantly undergoes fission and fusion to meet the cell’s bioenergetic needs (Rahman and Quadrilatero, 2021). These dynamic processes enable the mitochondria to adjust their structure and function in response to the cell’s energetic requirements (Fenton et al., 2021). This adaptability is crucial for maintaining cellular energy homeostasis and optimizing muscle performance (Flockhart et al., 2021). Exercise, particularly high-intensity and endurance training, has been shown to influence mitochondrial dynamics, enhancing their function and efficiency to support muscle performance (Casuso and Huertas, 2020; Smith et al., 2023).

The benefits of moderate exercise on overall health are well-established, with exercise regimens tailored to meet the specific needs of diverse populations, including individuals with various diseases, conditions, and age groups (Granata et al., 2018; Widmann et al., 2019). Understanding the impact of different exercise modalities on mitochondrial respiratory function and how these changes influence skeletal muscle performance is crucial for optimizing exercise recommendations across different demographic groups (Russomando et al., 2020). Mitochondrial respiratory function refers to mitochondrial electron transport system (ETS) activity and oxidative phosphorylation (OXPHOS), which are closely associated with exercise. Mitochondrial adaptations, such as increased synthesis of ETS proteins (i.e., mitochondrial biogenesis), are linked to improved metabolic health (Trewin et al., 2018). In certain subhealthy populations, such as those with obesity, exercise training improves mitochondrial respiration and presents potential target pathways for preventing lipotoxicity associated with type 2 diabetes in skeletal muscle (Mendham et al., 2021). In terms of skeletal muscle adaptations, the cellular stress induced by exercise and the subsequent metabolic signals that trigger mitochondrial biogenesis are closely linked to exercise intensity (Reisman et al., 2024). The general view is that high-intensity interval training (HIIT) is more effective than moderate-intensity continuous training (MICT) in improving aerobic capacity (Hwang et al., 2019), physical fitness, body composition (Hooshmand Moghadam et al., 2021), blood lactate clearance (Xie et al., 2024), mitochondrial function (Pengam et al., 2023), and maximal oxygen uptake (Milanovic et al., 2015) in individuals, as high-intensity exercise triggers a greater physiological response (MacInnis and Gibala, 2017). However, this view remains controversial in certain aspects (Chavanelle et al., 2017; Wewege et al., 2017; Oliveira et al., 2024). HIIT is not only time-effective but also requires much lower energy expenditure compared to traditional MICT, making it a more efficient option for individuals with time constraints (Thum et al., 2017). The relatively rapid rate at which mitochondrial content responds to training allows for relatively short-term studies of mitochondrial adaptations in humans (MacInnis and Gibala, 2017). We hypothesize that mitochondrial adaptations following HIIT and MICT may differ. HIIT may be more effective in certain contexts due to greater activation of specific kinases (e.g., AMPK and CaMKII) (Wang et al., 2024b), stronger metabolic signals (such as ATP turnover, accumulation of intracellular lactate, creatine, AMP, and ADP) (Skelly et al., 2021), and higher rates of mitochondrial biogenesis (MacInnis et al., 2017). To date, there have been no studies directly comparing the differential effects of two exercise modalities on mitochondrial dynamics. The specific exercise modality, the pathways and molecular mechanisms through which it influences mitochondrial morphology and dynamics, and whether these effects subsequently lead to distinct functional responses in skeletal muscle remain unclear.

Furthermore, understanding how improvements in mitochondrial respiratory capacity could benefit health and performance is essential, especially for individuals starting an exercise program. Mitochondrial adaptations are key to improving energy metabolism, endurance, and recovery, all of which contribute to enhanced athletic performance and metabolic health in both clinical and general populations.

Emerging research highlights the dynamic nature of mitochondrial location and function within cells, governed by fusion and fission events that create an extensive communicative network (Go et al., 2018; Antonicka et al., 2020; Flockhart et al., 2021; Rahman and Quadrilatero, 2021). Molecular events induced by various training modalities appear to initiate a cascade of adaptations, resulting in the remodeling of mitochondrial phenotypes (Pengam et al., 2023). Manipulating different exercise training variables can promote distinct and specific mitochondrial adaptations in skeletal muscle (Rahman and Quadrilatero, 2021; Dong and Tsai, 2023; Pengam et al., 2023). Thus, the regulation of mitochondrial morphological heterogeneity and the interplay between different aspects of mitochondrial dynamics by various exercises warrant further attention.

Given the uncertainty of the roles of MICT and HIIT in mitochondrial morphology, dynamics, and respiratory function, the aims of this study were to: (i) compare physiological and endurance performance measurements, as well as skeletal muscle mitochondrial respiratory function, including citrate synthase and complex I activities, before and after MICT or HIIT; (ii) comprehensively assess mitochondrial volume density and network morphology within myofibers using multi-dimensional approaches; (iii) preliminarily assess the mRNA levels of mitochondrial dynamics-related proteins in skeletal muscle, thereby exploring their relationship with mitochondrial respiratory function to gain deeper insights into the mechanisms of mitochondrial plasticity, adaptation and network remodeling in skeletal muscle.

## 2 Result

### 2.1 Six weeks of training enhance aerobic capacity, citrate synthase (CS) and complex I activities, with more pronounced effects from HIIT

**FIGURE 1:**
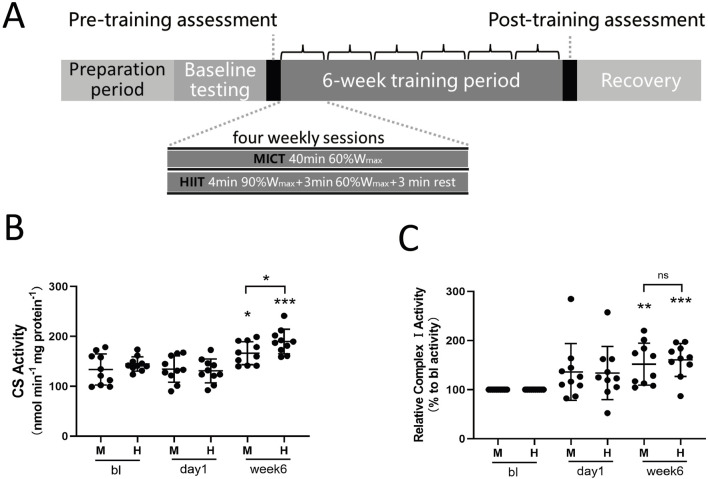
**(A)** Study Design: The shaded progress bar represents the training phases: preparation period, baseline testing (BL), and 6-week training period. Open bars indicate four sessions of either MICT or HIIT per week. HIIT consisted of four weekly sessions, each lasting 40 min, with four 4-min intervals at 90% of W_max_ followed by 3 min at 60% of W_max_, and 3 min of active recovery. MICT consisted of four weekly sessions, each lasting 40 min at 60% of W_max_. **(B)** Citrate Synthase Activity: Evaluation of citrate synthase (CS) enzyme activity in skeletal muscle mitochondria. **(C)** Complex I Activity: Evaluation of complex I activity in skeletal muscle mitochondria. Data are presented as the mean ± SD from three independent experiments, each performed in triplicate. Unless otherwise noted, *p < 0.05, **p < 0.01, ***p < 0.001 indicate statistical significance versus the BL group.

We recruited 20 healthy male participants with normal weight and no prior professional training. They underwent a 6-week training program that included either high-intensity interval training or moderate-intensity continuous training (see Table 1 for subject characteristics). The study design and training protocol are shown in Figure 1A. At the baseline stage, we determined the participants’ maximal workload (W_max_). Afterward, each group trained four times per week. The MICT group exercised continuously for 40 min at 60% of their W_max_, while the HIIT group performed four cycles of high-intensity intervals, each consisting of 4 min at 90% of W_max_ followed by 3 min at 60% of W_max_, with 3 min of active recovery between cycles. Changes in physiological and performance parameters are presented in Table 1. Six weeks of training significantly increased VO_2max_ and W_max_, indicating enhanced performance (Table 1). Specifically, VO_2max_ increased from 44.2 ± 8.8 to 49.0 ± 10.5 mL/min/kg in MICT group and from 44.4 ± 7.8 to 52.0 ± 11.2 mL/min/kg in the HIIT group. W_max_ increased from 225.2 ± 47.1 to 251.0 ± 44.4 W in the MICT group and from 223.8 ± 39.2 to 256.1 ± 46.2 W in the HIIT group. Training also resulted in an increase in citrate synthase activity (Figure 1B) and complex I activity (Figure 1C), which are considered rough markers of mitochondrial content and oxidative capacity. Among the two training modalities, HIIT demonstrated greater effectiveness compared to MICT in driving mitochondrial adaptations, as reflected by greater elevations in citrate synthase activity (HIIT:189.7 ± 24.6 vs. MICT:166.3 ± 22.8 µmol/min/mg protein, p = 0.041) (Figure 1B), while the increase in complex I activity was not statistically significant (HIIT:161% ± 34% vs. MICT:152% ± 43% to bl, p = 0.61) (Figure 1C). Notably, 24 h after either MICT or HIIT, neither mitochondrial Citrate synthase nor Complex I activity showed significant changes (Figures 1B,C).

**TABLE 1:** Participants’ physiological and endurance performance measurements.

	MICT		HIIT	
Measurement	Pre	Post	Pre	Post
Age	21.8 ± 2.2	-	22.1 ± 2.6	-
Height	173.1 ± 8.2	-	172.8 ± 9.3	-
Body mass	70.0 ± 15.3	69.7 ± 15.5	73.2 ± 13.3	71.4 ± 13.0
BMI	24.6 ± 2.5	24.5 ± 2.4	24.3 ± 2.8	24.1 ± 3.1
VO_2max_	44.2 ± 8.8	49 ± 10.5*	44.4 ± 7.8	52 ± 11.2*
W_max_	225.2 ± 47.1	251.0 ± 44.4*	223.8 ± 39.2	256.1 ± 46.2*

BMI, Body Mass Index [kg/m^2^]; VO_2max_, Maximal oxygen consumption [mL/min/kg]; Ẇ_max_, Maximal output power [Watts]; Values are mean ± SD., Measurements were taken before (pre) and after (post) the 6-week MICT, or HIIT; n = 10 for all analyses. *P-values <0.05 versus Pre group.

### 2.2 Six weeks of training increased mitochondrial volume density, with HIIT showing higher density, especially in intermyofibrillar mitochondria

**FIGURE 2:**
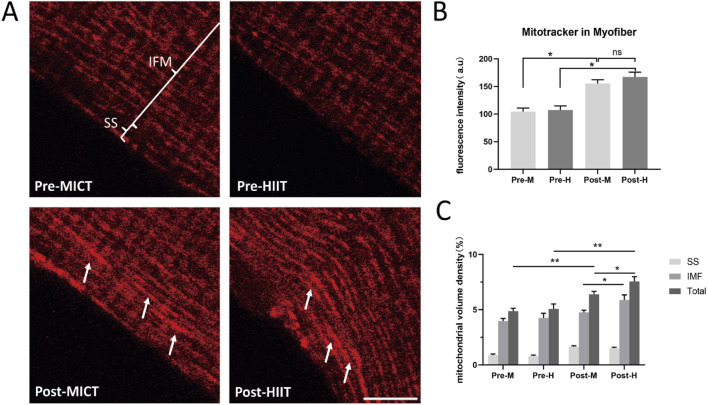
**(A)** Mitochondrial Fluorescence Images: Mitochondria in isolated myofibers were labeled with MitoTracker Orange. Pre-MICT: Before MICT, the annotations highlight the subsarcolemmal (SS) and intermyofibrillar (IMF); Pre-HIIT: Before HIIT; Post-MICT: After 6-week of MICT; Post-HIIT: After 6-week of HIIT. Scale bar = 10 μm. The white arrows indicate the regions with higher fluorescence intensity. Three independent experiments were conducted; representative images are shown. **(B)** Fluorescence intensity quantification. Fluorescence intensity of MitoTracker in the same area of isolated myofibers; **(C)** Volume density quantification. Mitochondrial volume density of SS, IMF, and total mitochondria (Total), as described in the “Materials and methods” section. Data are presented as the mean ± SD from three independent experiments, each performed in triplicate. *p < 0.05, **p < 0.01, ns: not statistically significant.

We cultured and isolated the myofibers from the vastus lateralis muscle biopsy samples taken from both groups before and after the 6-week training. The mitochondria were labeled with MitoTracker, and confocal microscopy imaging was conducted (Figure 2). Fluorescence analysis of fixed images revealed that the training intervention increased mitochondrial volume density in both groups, the white arrows indicate the regions with higher fluorescence intensity (Figures 2A,B). Although the HIIT training group exhibited a trend towards higher mitochondrial volume density compared to the MICT group, preliminary fluorescence intensity analysis indicated no statistically significant differences between the groups following training (p = 0.081) (Figure 2B). This may be due to the distinct yet potentially equal contributions of training intensity and duration to mitochondrial adaptations, both of which could influence mitochondrial content in different ways. Given the heterogeneity of mitochondria and the differential responsiveness of subsarcolemmal and intermyofibrillar mitochondria to various exercise modalities (Kras et al., 2018), we conducted a detailed analysis of mitochondrial volume density in subsarcolemmal mitochondria, intermyofibrillar mitochondria, and total mitochondria for both groups. This analysis showed that mitochondrial volume density in the HIIT group was higher than in the MICT group (p = 0.047), with intermyofibrillar mitochondria being the most affected (p = 0.045) (Figure 2C).

### 2.3 HIIT and MICT training altered the morphology and arrangement of the mitochondrial network in myofiber

**FIGURE 3:**
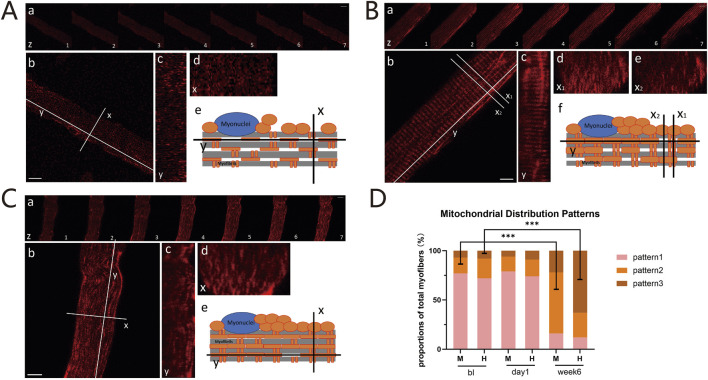
**(A)** Pattern 1: Sparse and Dispersed Punctate Mitochondria Network. (a) Z-axis scans with 1-micron intervals. Scale bar = 10 μm; (b) X-axis (I-bands, transverse) and Y-axis (contraction, longitudinal) of the myofibers. Scale bar = 10 μm; (c) Sagittal plane along the Y-axis; (d) Cross-sectional plane along the X-axis; (e) Schematic of mitochondrial distribution within myofibers. **(B)** Pattern 2: Grid-like Mitochondria Network with Robust Longitudinal Connections: Panels (a), (b), and (c) are the same as **(A)**. Cross-sections with dense (d) or sparse (e) mitochondria. Schematic diagram (f). **(C)** Pattern 3: Enhanced Longitudinally-oriented Mitochondria Network: Panels are the same as **(A)**. **(D)** Proportions of Three Mitochondrial Configurations: Proportions of these mitochondrial distribution patterns between the HIIT and MICT groups at different time points. ***p < 0.001 versus BL group.

The functional capacity of mitochondria is critically influenced by their cellular content, location, and network structure (Dong and Tsai, 2023). Therefore, by utilizing three-dimensional imaging that combines the xy-plane with the z-axis, we initially analyzed the distribution patterns of mitochondria within isolated myofibers. This analysis revealed significant differences in the overall arrangement of the mitochondrial network, which we classified into three distinct configurations: (i) Sparse and dispersed punctate mitochondria network: Mitochondria are sparsely distributed in a punctate pattern, aligning along both the I-bands (transverse axis) and the contraction axis (longitudinal axis) of the myofibers. They exhibit nearly equivalent amounts of parallel and perpendicular orientations, without forming distinct networks (Figure 3A). (ii) Grid-like mitochondrial network with robust longitudinal connections: Mitochondria form a grid-like network with distinct connections along the contraction axis and relatively uniform connections along the I-bands (Figure 3B). (iii) Enhanced longitudinally-oriented mitochondrial network: Mitochondria are densely connected along the contraction axis, while they are relatively sparse along the I-bands, resulting in the absence of a distinct grid-like structure (Figure 3C). We calculated the proportions of these three arrangement patterns at three time points: before training, 1 day after training, and 6 weeks after training in both groups. Before training, the first distribution pattern predominated (MICT: 77%, HIIT: 72%). At 1 day post-training, the mitochondrial distribution configurations remained unchanged (MICT: 79%, HIIT: 74%) (Figure 3D). After 6 weeks of training, significant changes were observed: the MICT group predominantly exhibited the second pattern (increased from 16% to 62% relative to bl, p < 0.001), whereas the HIIT group primarily showed the third pattern (increased from 8% to 63% relative to bl, p < 0.001) (Figure 3D). Additionally, there is a statistically significant difference between the two patterns in the two groups (M vs. H for both pattern 2 and pattern 3, with P < 0.001).

### 2.4 Mitochondrial dynamics-related mRNA showed activation of both fusion and fission 1 day post-training, but a trend towards promoting fusion and inhibiting fission after 6 weeks

**FIGURE 4:**
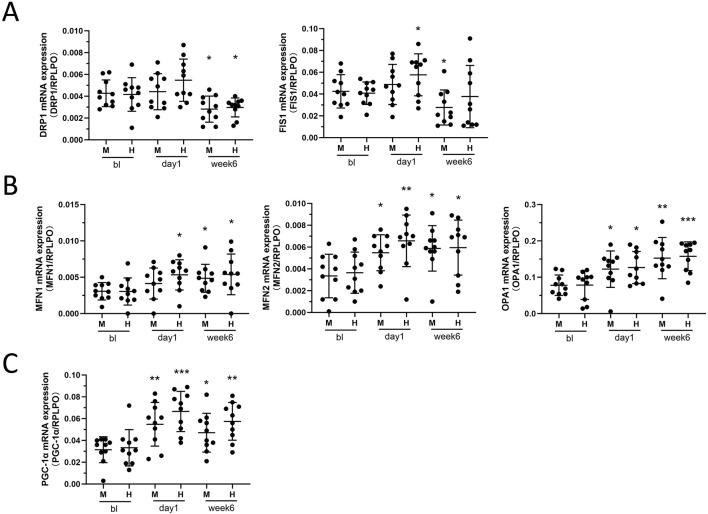
**(A)** Mitochondrial Fission: DRP1 and Fis1 mRNA expression between HIIT and MICT groups at different time points. **(B)** Mitochondrial Fusion: MFN1, MFN2, and OPA1 mRNA expression. **(C)** Mitochondrial Lifecycle Regulator: PGC-1α mRNA expression. Data are presented as mean ± SD of three separate experiments performed in triplicate. *p < 0.05, **p < 0.01, ***p < 0.001 versus BL group.

We found that the overall trend of mitochondrial fission protein showed an increase after 24 h of exercise, with a statistically significant increase in FIS1 observed only in the HIIT group (p = 0.0275) (Figure 4A). However, after 6 weeks of training, DRP1 decreased in two groups (M: p = 0.0154; H: p = 0.0475) (Figure 4A), with a significant decline in FIS1 observed in the MICT group (p = 0.0489), while the decline in FIS1 in the HIIT group was not statistically significant (p = 0.75) (Figure 4A). MFN1 and MFN2, which fuse the mitochondrial outer membrane (Fenton et al., 2021), as well as inner membrane fuser OPA1, increased after 1 day (24 h) and 6 weeks of HIIT and MICT (Figure 4B). Specifically, for MFN1, the increase at 24 h was significant in the HIIT group (p = 0.0185), but not in the MICT group (p = 0.1864). After 6 weeks, both groups showed significant increases (M: p = 0.0236; H: p = 0.0405) (Figure 4B). MFN2 showed significant increases in both groups at 24 h (M: p = 0.0181; H: p = 0.0069) and remained significant at 6 weeks (M: p = 0.0125; H: p = 0.0328) (Figure 4B). Similarly, OPA1 significant increases were observed at both 24 h (M: p = 0.0251; H: p = 0.0191) and 6 weeks (M: p = 0.0191; H: p = 0.0003) in both groups (Figure 4B). Peroxisome proliferator-activated receptor γ coactivator 1-alpha (PGC-1α), a potent transcription factor, plays a pivotal role in regulating mitochondrial biogenesis and oxidative stress (Abu Shelbayeh et al., 2023). Our findings indicate that PGC-1α and the mitochondrial fusion/fission proteins demonstrated similar expression profiles of change at the 24 h (M: p = 0.0053; H: p = 0.0005) and 6 weeks (M: p = 0.033; H: p = 0.0051) (Figure 4C).

## 3 Discussion

Skeletal muscle cells face increased energetic demands during exercise, yet the mechanisms governing mitochondrial remodeling to meet these metabolic challenges remain incompletely understood (Memme et al., 2021). Mitochondria, the primary energy producers in skeletal muscle cells, exhibit distinct adaptations in response to different training stimuli affecting mitochondrial dynamics signaling (Flockhart et al., 2021). This study contributes to the understanding of how skeletal muscle mitochondria adapt, undergo network remodeling, and regulate mitochondrial dynamics in response to training. Our findings highlight the differences between HIIT and MICT in mitochondrial adaptation. Both exercise modalities induced characteristic adaptive changes in the mitochondrial network, with MICT exhibiting a grid-like network and HIIT showing an enhanced longitudinally-oriented network (Figure 3). Furthermore, we observed that after 6-week training, the mRNA expression of genes involved in mitochondrial dynamics generally showed trend that promoted fusion and inhibited fission (Figure 4). These findings were further confirmed by the observed trends in mitochondrial network branching during live-cell imaging (Supplementary Figure S1).

Our 6-week training data confirm that a 6-week regimen of HIIT yields slightly superior improvements in citrate synthase (Figure 1B) and mitochondrial volumetric density (Figure 2C) compared to MICT. This result supports the prevailing view that HIIT elicits a stronger metabolic response than MICT: Higher ATP turnover, increased accumulation of intracellular lactate, creatine, AMP, and ADP, as well as greater activation of AMPK and CaMKII. Additionally, mitochondrial protein synthesis was greater following HIIT, further highlighting its superior impact on mitochondrial function (MacInnis and Gibala, 2017). HIIT demonstrating superior effectiveness in certain aspects may be directly related to mitochondrial dynamics, although the upstream and downstream interactions remain unclear, at least there appears to be some association. HIIT alternates short bursts of high-intensity exercise (80%–95% of maximum heart rate) with brief recovery periods. HIIT is known for its efficiency in enhancing cardiovascular fitness, boosting metabolism, and burning fat (Mueller et al., 2021). MICT involves sustained, moderate-level aerobic exercises such as jogging or cycling, typically performed at 60%–70% of maximum heart rate for 30–60 min. It improves cardiovascular health, muscle endurance, and metabolic function (Mueller et al., 2021). There is no consensus on whether MICT or HIIT is superior, as both exercise modalities affect various parameters differently (Mueller et al., 2021; Pengam et al., 2023; Oliveira et al., 2024; Wang et al., 2024a).

Mitochondrial dynamics, encompassing the processes of fission and fusion, are essential for maintaining both mitochondrial and cellular health (Chan, 2020). Mitochondrial fusion involves two distinct steps: the fusion of the outer membrane, which is orchestrated by mitofusin 1 (MFN1) and mitofusin 2 (MFN2), and subsequently, the joining of the inner membrane, aided by optic atrophy 1 (OPA1) (Hong et al., 2022). Conversely, mitochondrial fission, which results in smaller mitochondrial fragments, is predominantly driven by dynamin-related protein 1 (Drp1), fission protein 1 (Fis1), and mitochondrial fission factor (Mff) (Chen et al., 2023). Throughout the mitochondrial lifecycle, fusion events promote the formation of interconnected mitochondrial networks, while fission enables the selective elimination of damaged organelles. Fission generates smaller, fragmented mitochondria, which are then transported to different regions of the myofiber to meet local energy demands. At the same time, the fission process provides an opportunity for damaged mitochondria to be identified and cleared, typically through mitophagy (Leduc-Gaudet et al., 2021). The equilibrium between fission and fusion is crucial for maintaining a healthy and functional mitochondrial network (Leduc-Gaudet et al., 2021). When skeletal muscle are exposed to conditions that disrupt metabolic homeostasis, such as exercise or aging, the balance of mitochondrial regulation can shift, leading to either an increase in synthesis and fusion or, alternatively, in fission and mitophagy, depending on the specific circumstances (Memme et al., 2021). Research indicates that mitochondrial fusion markers are upregulated following exercise training, while mitochondrial fission remains unchanged in healthy individuals (Hood et al., 2011; Axelrod et al., 2019; Flockhart et al., 2021). For elderly individuals, as well as those with muscle atrophy and metabolic disorders, targeting mitochondrial dynamics holds significant potential as a therapeutic strategy for preventing and treating muscle atrophy, including through exercise therapy (Fealy et al., 2021; Leduc-Gaudet et al., 2021). Balancing mitochondrial dynamics is a feasible strategy against skeletal muscle atrophy. Diverse types of exercise regulate mitochondrial dynamics through distinct signaling and regulatory pathways, helping to prevent skeletal muscle atrophy (Lei et al., 2024). Therefore, our result on exercise modalities holds the potential to contribute to exercise therapies that balance mitochondrial dynamics.

In this study, examining the mRNA expression of mitochondrial fusion and fission genes revealed that 24 h after a single acute training session, both processes were simultaneously activated (Figure 4). However, after 6 weeks, there was a trend of enhanced mitochondrial fusion and inhibited fission (Figure 4). This was confirmed by time-lapse imaging (Supplementary Figure S1). Previous studies have confirmed that high-intensity aerobic interval training increases the OPA1/FIS1 ratio, indicating enhanced mitochondrial fusion, which is associated with improved mitochondrial respiration, insulin sensitivity, and Vo_2peak_ (Ruegsegger et al., 2023). Our 6-week study also promoted mitochondrial fusion, further reinforcing the findings from earlier research and highlighting the health benefits of HIIT. In conjunction with our result of citrate synthase and complex activities, as well as VO_2max_ and Ẇ_max_, we can infer that exercise can indeed enhance mitochondrial respiratory function in healthy individuals, promote mitochondrial fusion, and potentially improve muscle performance, thereby possibly benefiting overall health. Interestingly, PGC-1α, closely related to mitochondrial dynamics, showed a more significant increase at 24 h compared to 6 weeks of training (Figure 4C), likely related to rapid adaptation and response mechanisms triggered by acute exercise, as well as the adaptive responses to sustained exercise stimuli.

This study directly employed isolating myofibers to observe the mitochondrial networks. The connectivity between cellular structures plays a pivotal role in facilitating coordinated movements of ions, molecules, and proteins, which are fundamental to numerous cellular processes (Bleck et al., 2018). Visualizing the mitochondrial network within skeletal muscle fibers, starting with an analysis of mitochondrial morphology, guides the study of mitochondrial network dynamics. Myofibers are large, highly organized syncytial cells (Moore and Holzbaur, 2018). Although muscle cell culture models exist, they do not fully mature into differentiated myofibers (Demonbreun and McNally, 2015). Therefore, isolating mature myofibers directly from human skeletal muscle is the most effective method to observe mitochondrial networks (Demonbreun and McNally, 2015; Awad et al., 2023).

Improvements in skeletal muscle metabolism induced by exercise training are associated with enhanced mitochondrial network structures achieved through dynamic remodeling (Moore and Holzbaur, 2018; Fenton et al., 2021; Rahman and Quadrilatero, 2021; Romanello and Sandri, 2021). Research has demonstrated that muscle mitochondrial networks can adopt parallel, perpendicular, or grid-like configurations, influenced by the type of muscle fiber (Bleck et al., 2018). The size, orientation, and extent of structural and electrical connectivity within the muscle mitochondrial reticulum are aligned with the functional demands of the cell (Fenton et al., 2021; Romanello and Sandri, 2021). Our results indicate that training promotes tight connections within the skeletal muscle mitochondrial network and that MICT and HIIT have distinct effects on the morphology and arrangement of these networks (Figure 3). MICT exhibits a grid-like structure, while HIIT displays stronger longitudinally oriented network (Figure 3). Whether this is related to HIIT’s induction of enhanced mitochondrial oxidative capacity and biogenesis remains unclear. However, we can suggest that exercise leads to the formation of a tight mitochondrial network, and that HIIT, which triggers tighter longitudinal connections, may be associated with its stronger metabolic response. Previous studies have demonstrated that exercise induces mitochondrial elongation, with endurance training promoting the formation of more robust mitochondrial structures (Axelrod et al., 2019). Our findings extend beyond mitochondrial morphology, providing a crucial foundation for discussing the intricate relationships between mitochondrial shape, connectivity, and molecular exchange. This comprehensive perspective emphasizes the significance of structural integration within the mitochondrial network, illustrating how different training modalities, such as MICT and HIIT, uniquely impact these dynamics. However, the precise molecular alterations driving these structural and functional adaptations remain to be elucidated through further detailed investigation.

Due to mitochondrial heterogeneity, there are significant functional differences in mitochondria located at various positions within the cell (Bleck et al., 2018; Willingham et al., 2021). Intermyofibrillar mitochondria (IMF) account for 80%, while subsarcolemmal mitochondria (SS) account for 20% (Willingham et al., 2021). IMF primarily supplies energy for muscle contraction, while SS provides energy for membrane transport, maintaining membrane potential and cytoplasmic homeostasis (Koves et al., 2005; Ferreira et al., 2010). These two types of mitochondria also respond differently to exercise, with IMF mitochondria showing increased ATP production in obese individuals, an effect not significant in SS mitochondria (Koves et al., 2005; Willingham et al., 2021). The maintenance of the mitochondrial network structure largely depends on the facilitation of potential energy distribution between the IMF interactions (Diaz-Vegas et al., 2019). Comparing the volume density of IMF and SS separately, the results showed that although both increased after training, the total mitochondrial volume density after 6 weeks of HIIT was slightly higher than that of MICT, primarily due to an increase in IMF (Figure 2). Our findings theoretically align with the observed functional adaptations in IMF mitochondria in response to exercise. The increase in total mitochondrial volume density, primarily due to the rise in IMF, supports the distinct adaptive response of IMF mitochondria to high-intensity exercise (Lundby and Jacobs, 2016; Espinosa et al., 2023).

The findings of this study should be interpreted in light of several limitations. Firstly, the sample size is small, and only male subjects were included. Secondly, the study focuses on a limited range of mitochondrial respiratory function and mitochondrial dynamics proteins. Additionally, some data lack certain validations and should be viewed in light of the background, such as analyzing mitochondrial volume density without quantifying mitochondrial content and not performing quantitative analysis of live cell time-lapse imaging. These limitations stem from the fact that the primary goal of this study was to observe the effects of different exercise modalities on myofiber mitochondrial networks and to explore the potential molecular changes involved. Other factors that could potentially affect mitochondrial morphology, such as muscle fiber type differences due to gender, have been excluded. Essentially, this study is observational and exploratory in nature. Based on our findings, we can indeed draw the main conclusion that exercise modalities significantly influence the distribution of mitochondrial networks. Other measurements in this study present valuable avenues for future research. For instance, live cell time-lapse imaging, which could be used to label key mitochondrial fusion and fission proteins in live cells and explore their correlation with mitochondrial distribution patterns. Combined with *in vitro* exercise models (Chen et al., 2019), it may be possible to visualize changes in mitochondrial network distribution in response to exercise stimuli.

Overall, the results highlight the role of MICT and HIIT in regulating skeletal muscle mitochondrial network dynamics, balancing morphology, fusion, and fission. Our data, in conjunction with previous studies, demonstrate that HIIT has superior effectiveness in mitochondrial biogenesis and volume density, making it a viable alternative to MICT. Moreover, we provide further insights into how different exercise regimens regulate the arrangement of the mitochondrial network within myofibers. The distinct characteristics of skeletal muscle mitochondrial network arrangement, orientation, and density in HIIT and MICT are consistent with the functional demands of energy metabolism. More detailed studies to elucidate the mechanisms driving exercise-induced mitochondrial network adaptive remodeling events using imaging-based approaches are underway.

## 4 Materials and methods

### 4.1 Participants and ethics approval

Twenty healthy men, aged 18–25 years, volunteered for this study, with their physiological and performance parameters detailed in Table 1. The participants were moderately trained, having engaged in less than 4 h of unstructured aerobic activity per week for 6 months prior to the study. None of the participants regularly participated in cycling-based sports, and all were non-smokers and free of medication during the study. Before the study began, the purpose, design, and associated risks were thoroughly explained to the participants, who then provided written informed consent. Health status was verified through medical history, routine physical examination, and standard blood and urine tests. Throughout the entire study period, all participants followed the same meal plan for breakfast, lunch, and dinner. The study procedures were approved by the ethical committee of Hunan Provincial Sports Vocational College (KYGS2023-24) in accordance with the Declaration of Helsinki. All exercise training and physical tests were conducted at the Hunan Sports Rehabilitation Training Center (HSRTC), Changsha, Hunan, China. The basic experiments were performed at Hunan Provincial Key Laboratory of Pediatric Orthopedics, Changsha, Hunan, China.

### 4.2 Study design, training and testing

The experimental design consisted of (i) Preparation and familiarization period: Participants were instructed to avoid vigorous exercise for 48 h before each performance test, abstain from alcohol and exercise for 24 h before testing, and refrain from food and caffeine for 2 h prior to each test. Tests were conducted at the same time each day to minimize circadian rhythm variations. Each participant repeated the familiarization procedure before baseline measurements. (ii) Baseline testing: Participants performed an incremental cycling test to volitional fatigue on an electronically braked cycle ergometer (Merach, Hangzhou, Zhejiang, CN) equipped with dual-sided pedal-based power meters (Rally RK200, Garmin, Shanghai, CN) to determine maximal workload (W_max_). Maximal oxygen uptake (VO_2max_) was measured using an athletic monitoring smartwatch (TruSport Huawei, Shenzhen, CN). The same equipment was used throughout the subsequent experimental sessions. (iii) Experimental Intervention: Participants were randomly assigned to either high-intensity interval training (HIIT) (N = 10) or moderate-intensity continuous training (MICT) (N = 10) for 6 weeks. Each group trained four times per week. The MICT group exercised continuously for 40 min at 60% of their W_max_, while the HIIT group performed four cycles of high-intensity intervals, each consisting of 4 min at 90% of W_max_ followed by 3 min at 60% of W_max_, with 3 min of active recovery between cycles. All training sessions were conducted under supervision.

### 4.3 Skeletal muscle biopsies

Subjects arrived at the laboratory in the morning after an overnight fasting period (beginning at 22:00). Following 15 min of supine rest, the biopsy procedure commenced. Skeletal muscle biopsies were taken from the vastus lateralis muscle of the dominant leg at three time points: baseline, day 1 (24 h after the first training session), and after 6 weeks of training (24 h following the final training session). Samples were collected under local anesthesia (1% lidocaine) of the skin and superficial muscle fascia, using the Bergström technique with a needle modified for suction. The biopsies were immediately dissected free of fat and connective tissue and divided into sections for further processing: (i) live and fixed imaging; (ii) complex I activity, citrate synthase activity, and mRNA expression (frozen in liquid nitrogen). All samples were labeled with codes unknown to the investigators.

### 4.4 Citrate synthase activity and chain respiratory complex I activity

Citrate synthase activity was measured in muscle lysates using a commercial assay kit (MAK193, Sigma-Aldrich, St. Louis, MO) following the manufacturer’s protocol. All activities were normalized to mg of total protein. Chain respiratory complex I activity was assessed with a MitoTox Complex I OXPHOS activity assay kit (ab109903, Abcam, Cambridge, United Kingdom), according to the manufacturer’s instructions. The results are presented as the relative complex I activity, normalized to the baseline.

### 4.5 Myofibers isolation and imaging

Muscle bundles were incubated with collagenase in DMEM + BSA at 37°C for 2 hours, then transferred to Ringer’s solution for trituration (Demonbreun and McNally, 2015). A 1 mL pipette was used to triturate the bundles until most fibers dissociated, with intact fibers settling in the solution. The fibers were then plated onto a laminin-coated glass dish for 15 min. The Ringer’s solution was replaced with myofiber culture medium (DMEM/Ham’s F10, 20% FBS, 1% penicillin-streptomycin, 1% chicken embryonic extract, 2.5 ng/mL bFGF) and cultured at 37°C, 5% CO_2_, for 5–24 h. Prior to imaging, fibers were incubated with MitoTracker Orange (Invitrogen, Carlsbad, CA, United States) at 37°C for 45 min, as per the manufacturer’s instructions.

For the live imaging experiments, the myofibers were immersed in a solution consisting of 150 mM NaCl, 5 mM KCl, 2 mM CaCl_2_, 1 mM MgCl_2_, 10 mM HEPES-NaOH (pH 7.4), and 5.5 mM d-glucose. For fixed imaging, myofibers were washed with PBS and fixed for 20 min with 2% paraformaldehyde in PBS containing 0.1% Triton X-100, then washed and blocked in PBS containing 5% CS and 1% BSA at room temperature. Myofibers were imaged on a Leica SP8 confocal microscope using the confocal mode with a 63× oil immersion/1.4 NA objective. A wavelength of 555 nm was used to excite MitoTracker. Gain and offset were set to values that prevented saturated and empty pixels. During the imaging process, both 2D xy-plane sections and 3D xyz-stacks (adding the z-axis, depth interval is 1 micron) were captured to obtain the most comprehensive visualization. Experiments in single myofibers were designed to alternate imaging at 10-s intervals (time interval 10s) and exposure for a total of 20–25 min (120–150 frames). Movie files and images of individual and merged time-lapse data were generated for further analysis. For each biopsy sample, 3-5 viable myofibers were selected for live imaging, and more than 10 for fixed imaging. Representative xy-plane images, xyz-stack images, and time-lapse images are presented.

### 4.6 Mitochondrial volume density

15–20 images per biopsy were acquired for analysis using Fiji ImageJ software (NIH, Bethesda, MD, United States). The images were preprocessed by converting to grayscale, subtracting background, and enhancing contrast. Thresholding was applied to segment mitochondria, followed by using the Analyze Particles tool to measure mitochondrial areas (Medeiros, 2008; Fogarty et al., 2021). The total mitochondrial volume and total muscle fiber volume were calculated by summing the areas of mitochondria and muscle fiber, respectively. Mitochondrial volume density (MitoVD) was estimated by dividing the total mitochondrial volume by the total muscle fiber volume. Mitochondria were categorized as either intermyofibrillar (IMF) or subsarcolemmal (SS) based on their location. IMF mitochondria were identified between myofibrils, while SS mitochondria were identified just beneath the sarcolemma, defined as those located within approximately 3 μm from it. The boundary between IMF and SS mitochondria is defined by the distinct morphological characteristics in each image. Specific MitoVD (IMF or SS) was calculated by dividing the volume of either IMF or SS mitochondria by the total muscle fiber volume.

### 4.7 Mitochondria distribution pattern analysis

10–15 complete, well-attached muscle fibers with uniform MitoTracker staining per biopsy were acquired for analysis. The mitochondrial morphology was analyzed using the MiNA plugin for ImageJ (https://github.com/StuartLab/MiNA) (Ljosa and Carpenter, 2009). First, we used the “skeletonize” function to generate a skeleton image of the mitochondrial network within the muscle fibers. Based on the skeleton, mitochondrial distribution patterns were visually classified into punctate, grid-like, and longitudinally-oriented patterns. This initial classification was done by three independent researchers, who each performed three repetitions to obtain the initial data. Next, we applied the MiNA plugin to differentiate mitochondrial networks (networks) and individual mitochondria (individuals) based on the binary skeleton image. We then calculated the number of individuals or networks, the mean branch length and the length standard deviation. The first distribution pattern, Sparse and dispersed punctate mitochondria, is characterized by a predominance of individual mitochondria. In the second pattern, Grid-like mitochondrial network, mitochondria are primarily distributed along the transverse axis, resulting in a higher number of branches with shorter and more uniform lengths. The third pattern, Enhanced longitudinally-oriented mitochondrial network, features mitochondria of varying lengths, with both individual and network present. The length standard deviation is larger in this pattern. We used this approach to validate the initial data and calculate the final percentages for each distribution pattern.

### 4.8 RNA extraction and real-time PCR analysis

RNA was isolated from myofibers utilizing TRI reagent (Thermo Fisher Scientific), followed by the synthesis of cDNA using a Transcriptor First Strand cDNA synthesis kit along with oligo-dT primers (Roche, Basel, Switzerland). Subsequently, quantitative real-time PCR (qRT-PCR) was conducted using SsoAdvanced Universal SYBR Green Supermix, and the results were analyzed with a Bio-Rad CFX Connect System (Bio-Rad Laboratories Inc., Hercules, CA, United States). The relative expression levels of the genes of interest were determined through the 2^−ΔCT^ method, employing reference genes for normalization. The following primers for qRT-PCR analyses were employed; for MFN1, 5ʹ-TGT TTT GGT CGC AAA CTC TG -3ʹ and 5ʹ- CTG TCT GCG TAC GTC TTC CA -3ʹ; for MFN2, 5ʹ- ATG CAT CCC CAC TTA AGC AC -3ʹ and 5ʹ- CCA GAG GGC AGA ACT TTG TC -3ʹ; for OPA1, 5ʹ- GTG TGG GAA ATT GAT GAG TAT ATC G-3ʹ; 5ʹ- GCA CTC TGA TCT CCA ACC AC; for Fis1, 5ʹ- CCG GCT CAA GGA ATA TGA AA-3ʹ and 5ʹ- ACA GCC AGT CCA ATG AGT CC-3ʹ; for DRP1, 5ʹ- TTA CGG TTC CCT AAA CTT CAC G-3ʹ; 5ʹ- GTC ACG GGC AAC CTT TTA CGA-3ʹ; for PGC-1α, 5ʹ- TCA GTC CTC ACT GGT GGA CA 3ʹ and 5ʹ- TGC TTC GTC GTC AAA AAC AG -3ʹ; for RPLP0, 5ʹ-GGA AAC TCT GCA TTC TCG CT-3ʹ and 5ʹ-GCA AGT GGG AAG GTG TAA TCC-3ʹ.

### 4.9 Statistical analysis

Statistical analyses were performed using GraphPad Prism version 10 (GraphPad Software Inc.). In this study, the mitochondrial distribution pattern percentages were analyzed using Student’s t-test. All other data were analyzed using Two-way ANOVA with Tukey’s multiple comparisons test, unless otherwise indicated in the figure or table legend. The factors in the Two-way ANOVA included the training modalities (HIIT or MICT) and the time points (baseline, day 1, week 6). Data are presented as mean ± SD from three independent experiments, each conducted in triplicate. A p-value <0.05 was considered statistically significant unless otherwise specified.

## Data Availability

The datasets presented in this study can be found in online repositories. The names of the repository/repositories and accession number(s) can be found below: https://figshare.com/s/2ec3ea76ad8fed482d64.
