# Supplementary material for: Effects of high-intensity interval training and moderate-intensity continuous training on mitochondrial dynamics in human skeletal muscle
Source: Front Physiol. 2025 Apr 17;16:1554222. doi: 10.3389/fphys.2025.1554222 (PMC12043657; doi:10.3389/fphys.2025.1554222)
Supplement: Supplementary file 1 [file Table1.docx]

**Figure S1:** **A.** **Mitochondrial Network Dynamics - Pattern 1. (**a) Isolated muscle fiber with the area to be enlarged indicated by a white box; (b) Time-lapse images of the enlarged area from panel (a), with a 10-second interval. Red circles indicate mitochondrial fusion or fission. The white arrows indicate the process of mitochondrial fusion, the yellow arrows indicate mitochondrial fission. Scale bar = 10 μm. **B.** **Mitochondrial Network Dynamics - Pattern 2:** Panels are the same as A. **C.** **Mitochondrial Network Dynamics - Pattern 3:** Panels are the same as A.

**Mitochondria in myofibers tend to fuse along the longitudinal axis post-training.**

Given that the mitochondrial network in skeletal myofibers undergoes continuous dynamic changes through fusion and fission, we conducted live-cell time-lapse imaging of in vitro cultured myofibers from skeletal muscle biopsy before and after training. We recorded the dynamic changes of the mitochondrial network in real-time. Live-cell time-lapse imaging observations showed that the mitochondrial network branches undergo continuous fusion and fission, a dynamic process that maintains a balanced distribution pattern of the network. From the limited live-cell imaging experiments we found that before and after MICT or HIIT, this dynamic balance in mitochondrial network distribution appeared to be consistent with the results from fixed imaging. This dynamic behavior of the mitochondrial network supports our previous conclusion that training promotes the expression of mRNA associated with mitochondrial fusion. Although both HIIT and MICT training result in similar fusion events, they exhibit differences in their respective spatial arrangement patterns.
