# Supplementary figures and images for: Effects of high-intensity interval training and moderate-intensity continuous training on mitochondrial dynamics in human skeletal muscle
Source: Front Physiol. 2025 Apr 17;16:1554222. doi: 10.3389/fphys.2025.1554222 (PMC12043657; doi:10.3389/fphys.2025.1554222)

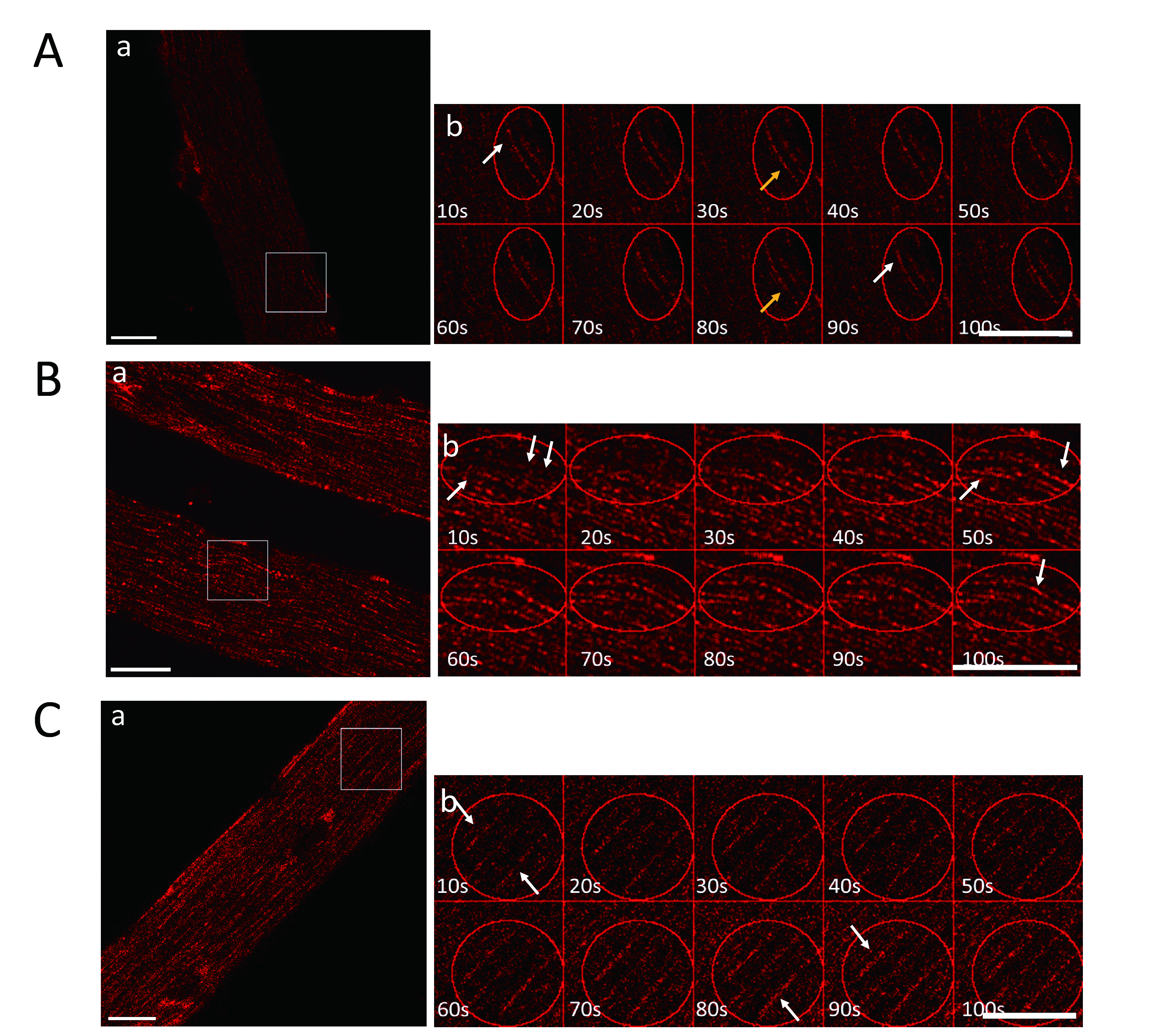

Supplement: Supplementary file 2 [file Image1.jpeg]
